# A role for two-pore potassium (K2P) channels in endometrial epithelial function

**DOI:** 10.1111/j.1582-4934.2012.01656.x

**Published:** 2013-01-11

**Authors:** Suraj K Patel, Leigh Jackson, Averil Y Warren, Pratibha Arya, Robert W Shaw, Raheela N Khan

**Affiliations:** a Academic Division of Obstetrics & Gynaecology, University of NottinghamDerby, UK; b Department of Obstetrics & Gynaecology, Royal Derby HospitalDerby, UK

**Keywords:** endometrium, potassium, channel, pH, proliferation, menstrual cycle

## Abstract

The human endometrial epithelium is pivotal to menstrual cycle progression, implantation and early pregnancy. Endometrial function is directly regulated by local factors that include pH, oxygen tension and ion concentrations to generate an environment conducive to fertilization. A superfamily of potassium channels characterized by two-pore domains (K2P) and encoded by *KCNK* genes is implicated in the control of the cell resting membrane potential through the generation of leak currents and modulation by various physicochemical stimuli. The aims of the study were to determine the expression and function of K2P channel subtypes in proliferative and secretory phase endometrium obtained from normo-ovulatory women and in an endometrial cancer cell line. Using immunochemical methods, real-time qRT-PCR proliferation assays and electrophysiology. Our results demonstrate mRNA for several K2P channel subtypes in human endometrium with molecular expression of TREK-1 shown to be higher in proliferative than secretory phase endometrium (*P* < 0.001). The K2P channel blockers methanandamide, lidocaine, zinc and curcumin had antiproliferative effects (*P* < 0.01) in an endometrial epithelial cancer cell line indicating a role for TASK and TREK-1 channels in proliferation. Tetraethylammonium- and 4-aminopyridine-insensitive outwards currents were inhibited at all voltages by reducing extracellular pH from 7.4 to 6.6. Higher expression of TREK-1 expression in proliferative phase endometrium may, in part, underlie linked to increased cell division. The effects of pH and a lack of effect of non-specific channel blockers of voltage-gated potassium channels imply a role for K2P channels in the regulation of human endometrial function.

## Introduction

The adult human endometrium is central to reproductive success undergoing monthly cyclical renewal in preparation for implantation. During the proliferative phase, regeneration of the endometrium involving increased proliferation of both stromal and epithelial cells occurs principally in response to oestradiol 17β. Following ovulation, progesterone converts the epithelium to a secretory phenotype, endometrial proliferation ceases and a transient receptive state, ‘the window of implantation’, is induced. During this brief period, electrolyte balance of the intrauterine fluid is crucial in ensuring apposition and attachment of the blastocyst to the luminal epithelium. Indeed, fluid shifts and oedema occur in the endometrium throughout the perimplantation period [1], yet comparatively little is known or understood regarding the mechanisms generating ionic gradients across the endometrium.

The ionic composition of intrauterine fluid differs from normal serum in having a higher potassium (K^+^) concentration [2] and is supported by evidence from *in vitro* ion measurements in supernatants from cultured human endometrial epithelium [3]. Interestingly, a K^+^-rich intrauterine environment has been shown to promote both sperm fertilizing capacity and blastocyst cleavage [4]. Thus, critical aspects of fertilization, implantation and early pregnancy are dependent on an appropriate ionic microenvironment which is in part determined by ion channel activity.

Ion channels provide a specialized conduit for the rapid transfer of ions across the lipid bilayer. Their specific roles in endometrial epithelia, in common with those in epithelia of other organs, *e.g*. kidney, airways, gastrointestinal tract, include electrolyte homeostasis, fluid/nutrient secretion and setting of the resting membrane potential [5]. A limited amount of information on ion channels within the human endometrium indicates the presence of an amiloride-sensitive sodium channel (ENaC) that mediates epithelial sodium absorption [3] as well as a cystic fibrosis transmembrane regulator (CFTR) involved in anion flux [6]. Gene profiling by microarray analysis has also identified trends in endometrial channel expression with specific phases of the menstrual cycle. For example, Kao *et al.,* [7] demonstrated a marked up-regulation of cDNA for the sulfonylurea receptor (a key accessory protein of the ATP-sensitive K^+^ channel) and the L-type calcium channel during the window of implantation. The gene *KCNJ2* (encoding an inwardly rectifying K^+^ channel, KIR2.1) has been shown to be highly expressed in proliferative phase endometrium along with *KCNG1* (encoding Kv6.1) and the epithelial sodium channel, *SCNN1A* gene for ENaC [8].

In recent years, the K2P channel superfamily, distinguished from the classical voltage- and receptor-gated K^+^ channel families by the presence of two pores arranged in tandem between four transmembrane domains, has achieved prominence. Channels belonging to the mammalian K2P family, first reported following the discovery of TWIK-1 [9], are gated by a range of physicochemical stimuli that include pH, oxygen tension and stretch [10, 11]. The channels include TWIK-1 [9], TWIK-2 [12], TREK (TWIK related) [13], TRAAK [14] and TASK [15] where TWIK-1 is a weak, inwardly rectifying channel, TRAAK channels are activated by arachidonic acid whereas TASK channels are acid sensitive [15].

Given the emerging significance of K2P channels in maintaining resting membrane potential and their coupling to cellular function, we propose that K2P channels in the human endometrium may be of crucial significance where slight perturbations locally in lipid regulation, ion transport, pH or oxygen tension could jeopardize the ambient conditions necessary for successful fertilization and implantation. The objectives of our study were therefore to determine the major K2P channel subtypes expressed in the human endometrium, their relationship to the menstrual cycle and their roles in endometrial physiology.

## Material and methods

### Patient inclusion criteria and tissue collection

Ethical approval for this study was granted by the Lincolnshire Research Ethics Committee (study no. 03/2/39). Endometrial tissue biopsies were collected from fully informed patients who provided written consent prior to clinical procedures being undertaken. The research described was undertaken in accordance with the World Medical Association Declaration of Helsinki. Criteria for recruitment were non-pregnant women aged between 18 and 40 years (mean 32.2 ± 4.7; *n* = 49), admitted for gynaecological investigation (pelvic pain, menorrhagia) or laparoscopic sterilization and with regular (28–34 days) monthly cycles. Endometrial samples collected from days 5–11 were assigned to the proliferative phase (*n* = 31) whereas secretory phase endometrium was designated as such based on a luteal phase progesterone level of >13 nmol/l (*n* = 11). In cases where progesterone levels were not available (for example, laparoscopic sterilizations), the secretory stage was defined as post-day 16 of the cycle, determined from the date of the last menstrual period (*n* = 7). Women diagnosed with diabetes, endometriosis, fibroids, polycystic ovarian syndrome, cancer or amenorrhea were excluded from the study as were those who had been on any form of hormonal contraception up to 1 month before recruitment to the study.

Endometrial biopsies obtained by pipelle sampling were placed immediately in Dulbecco's modified Eagle's Medium (DMEM, Invitrogen, Paisley, UK) and supplemented with 1% penicillin/streptomycin. Samples were washed in ice-cold sterile phosphate-buffered saline and dispersed no longer than 24 hrs after collection or snap frozen within 10 min. for downstream analyses of molecular and protein channel expression.

Cell line: The Ishikawa endometrial cell line (99040201), derived from an endometrial adenocarcinoma of a 39-year-old woman, was obtained from the European Collection of Cell Culture (Sigma-Aldrich, Dorset, UK).

### Primary endometrial cell culture

Fresh endometrial biopsies (50–500 mg) were finely chopped and enzymatically digested with collagenase (1 mg/ml; Sigma-Aldrich) in calcium/magnesium-free Hanks Balanced Salt solution (HBSS) in a 37°C water bath for a maximum of 45 min. If the tissue sample contained obvious amounts of blood (particularly early and late menstrual stage samples), then the preparation was carefully layered onto a 60% Percoll (Invitrogen, Paisley, Scotland) solution and centrifuged at 800 r.p.m. for 30 min. at 4°C to remove erythrocytes.

Following centrifugation, cells were transferred to a fresh tube containing HBSS then passed through a 40 μm nylon cell strainer (BD Falcon, Oxford, UK) to separate stromal and epithelial cells. The former, owing to their smaller size, passed through the strainer whereas the larger glandular cells were retained. Both cell fractions were separately passed through the cell strainer a second time to further enrich the two cell preparations, washed again in HBSS then centrifuged at 50 *g* for 7 min. The final step involved resuspending the cells in DMEM supplemented with 10% heat-inactivated foetal bovine serum (Sigma-Aldrich) followed by incubation at 37°C in a humidified incubator containing 5% CO_2_ in air. Media was changed every 2–3 days for a maximum of 14 days or two passages.

### RNA extraction and cDNA synthesis

Total RNA was extracted from either snap-frozen endometrial biopsies (50–200 mg) or ∼5–9 × 10^6^ cells of the cultured Ishikawa cell line, using TriZol reagent according to the manufacturer's instructions. Only RNA that resulted in an A260/280 ratio of 1.8–2.0, and displayed clearly distinguishable 18s and 28s rRNA bands in an agarose gel was reverse transcribed to generate cDNA. Synthesis of cDNA was carried out with SuperScript II RNase Reverse Transcriptase (200 units/μl, Invitrogen) and Oligo (dT)_12-18_ primers (Invitrogen) at 42°C with 1 μg of total RNA as template, in a final volume of 20 μl. Negative controls for the reverse-transcription reaction were prepared by omitting the RT enzyme.

For conventional end-point PCR, 100 ng of cDNA was amplified following addition to a 25 μL mastermix containing dNTPs, Platinum® Taq DNA polymerase (Invitrogen) enzyme and appropriate forward and reverse primers for the desired K2P target gene. Amplicons were visualized under UV light following separation through a 2% agarose gel containing ethidium bromide.

### qRT-PCR

Quantitation of TWIK-2 and TREK-1 mRNA was carried out by real-time PCR (qPCR) using SYBR I green chemistry on an MJ Chromo 4 thermal cycler (Opticon Monitor 3.1.32 software, BioRad, Hemel Hempstead, UK). Approximately 1 ng/μl of endometrial cDNA was added to Platinum® SYBR® Green qPCR Supermix-UDG (Invitrogen) and primers (maximum concentration 250 nmol) in a 25 μl reaction. The PCR protocol comprised the following steps: 50°C for 2 min., 95°C for 10 min., followed by 40 cycles of 95°C for 15 sec. and 60°C for 60 sec. for annealing and extension. The cycle terminated with melt-curve analysis of the amplicon in 1°C increments (65–96°C) to exclude contamination from primer dimers and to confirm amplification of a single, specific transcript. All conditions and methods for TREK-1 qPCR were as for TWIK-2, with the exception of an annealing temperature of 62°C for TREK-1. Standard curves were generated from serially diluted endometrial cDNA and the target transcripts quantitated by normalizing expression relative to an endogenous reference gene, in this case, hypoxanthine-guanine phosphoribosyltransferase (HPRT). Efficiency, calculated from standard curves, varied from 90% to 104% for endometrial cDNA over a threefold dilution series. Exon-spanning primer pairs used for conventional RT-PCR and qRT-PCR are shown in Table 1. The same TWIK-2 and TREK-1 primers were used in conventional and qRT-PCR.

**Table 1:** Primers used for RT-PCR and qRT-PCR for K2P channels in human endometrium

Gene	Protein	Sequence 5-3′	Accession number
*KCNK2*	TREK-1	TCAGCAAATAGTGGCAGCAA AGTGCCAGCAAAGAAGAAGG	NM_001017424
*KCNK10*	TREK-2	GAGAAGCGCTCTGTCTTTGC TCTTTGGTGTCCGTGGGTAT	NM_138317.1
*KCNK4*	TRAAK	GGAGAAGGAGCAGCCACTG GCTCTGCGTATCCGAGGACT	NM_03310.2
*KCNK1*	TWIK-1	GCGCAGTGGTCTTCTCCTC GAAGTCCCAGTTCCAGTTGC	NM_002245
*KCNK6*	TWIK-2	CAAGGTGCTGGTCACAGTCT ATGGAAGCGTAGTCGGTGTG	NM_004823
*KCNK3*	TASK-1	AACGCCGAGGACGAGAAG CTCTTGTACCACAGGCACGA	NM_002246
*KCNK5*	TASK-2	GGAAGAAGGCCATGAAGACA AGTGCTGGTGAAGGTGGACT	NM_003740
*KCNK9*	TASK-3	TCGTCCTCAGGTTCTTGACC CCCAGGAGAGATGGAGCTAA	NM_016601
*KCNK17*	TASK-4/TALK-2	CTCGCCTACCTGGCTTACCT CGGTCCAGACACGTGAAGTT	NM_031460
*KCNK16*	TALK-1	GCTGGCCTATGTCTGCTACC ACTGGTCCAGGCAGGTGTAG	NM_032115
*KCNK13*	THIK-1	TGGATCCTGAGGAAAATGGA TTGGCCATCTCAGACAGTTG	NM_022054
HPRT1	HPRT	TGACCTTGATTTATTTTGCATACC TGAGGAATAAACACCCTTTCCA	NM_000194
	β-actin	CATGTACGTTGCTATCCAGGC CTCCTTAATGTCACGCACGAT	NM_001101
	GAPDH	CATGAGAAGTATGACAACAGCCT AGTCCTTCCACGATACCAAAGT	NM_002046
	β_2_M	GCTATCCAGCGTACTCCAAA GAAAGACCAGTCCTTGCTGA	NM_004048

### Immunohistochemistry

Endometrial biopsies were cleared of mucus or clots, snap-frozen and embedded in OCT medium. Approximately 5 μm thick frozen serial sections were mounted on aminopropyltriethoxysilane (APES)-coated slides and air dried. Endogenous peroxidize activity was blocked with 3% (w/v) aqueous hydrogen peroxide and slides washed with water. Sections were then stained for 1 hr with a primary antibody raised to E-cadherin (BD Biosciences, Oxford, UK) and washed thereafter. After incubation with a biotinylated secondary antibody (DAKO Labs Ely, Cambs, UK) and further washing, sections were exposed for 8 min. to the avidin–biotin complex (ABC; Vector Laboratories, Peterborough, UK) followed by 3′3-diaminobenzidine for detection of bound antibody. Thereafter, slides were rinsed and counterstained with Harris's haematoxylin before viewing under light microscopy (Zeiss Axiovert, Carl Zeiss, Jena, Germany).

### Immunofluorescence

Cells cultured to a maximum of two passages were processed as previously described [16]. Briefly, endometrial cells on glass coverslips were fixed in 4% paraformaldehyde for 10 min. followed by permeabilization with 0.1 M Igepal in PBS. Following block of non-specific sites with 5% goat serum, cells were incubated with anti-TWIK-2 (1 in 50) or anti-TREK-1 (1 in 50) antibody following exposure to FITC-conjugated secondary antibody. Cells were subsequently viewed using either an epifluorescence (Zeiss Axiovert) microscope or a Zeiss Axiovert 100 microscope with an LSM 510 confocal scan head (Carl Zeiss, Jena, Germany) and a plan-Apochromat ×63 oil immersion objective lens. For control wells, cells were incubated with control IgG at the same dilution instead of the primary antibody.

### Western blotting of channel proteins

Individual, unpooled snap-frozen endometrial samples (∼200 mg) were processed in homogenization buffer (300 mM sucrose, 25 mM Tris-base, 10 mM monothioglycerol, 1 mM EDTA, 1% Igepal) and protease/phosphatase inhibitor cocktail. Following centrifugation of the tissue preparation at 1000 g for 10 min. at 4°C, the supernatant was removed and centrifuged again at 14,000 × *g* for 60 min. Solubilization of the pellet was carried out overnight at 4°C in solubilization buffer (20 mM, Tris-base pH 7.5, 10 mM, EDTA 120 mM NaCl, 50 mM KCl, 2 mM dithiotreitol, Igepal 2.5% and a cocktail of protease/phosphatase inhibitors). The protein concentration of all samples was determined using the bicinchoninic acid (BCA) assay. Samples were added to sample buffer (1 mM Tris-HCl, 10% SDS, 0.05% bromophenol blue and β-mercaptoethanol) and boiled at 95°C for 5 min. after which 40 μg protein per lane was loaded and resolved by SDS-PAGE on 10% gels. Following electrophoretic transfer of proteins onto nitrocellulose membranes, blocking of non-specific sites was performed for 2 hrs with 5% Marvel in TBS-T followed by washing in TBS-T. Membranes were subsequently incubated overnight at 4°C in anti-TREK-1 (1 in 500) or anti-TWIK-2 antibody (1:2500 dilution) prepared in TBS-T containing 3% Marvel. After washing the blots, alkaline-phosphatase-conjugated secondary antibody (goat antimouse, 1:800 dilution) was applied for 2 hrs. Immunolabelled protein bands were visualized after washing with TBS and exposure to chemiluminescent substrate (ImmunStar, BioRad, Herts, UK) and protein densities quantified after deducting non-specific background. Molecular weights of target proteins were determined by running Precision Plus molecular weight markers (BioRad) alongside. Channel antibodies were purchased from Caltag-Medsystems Ltd (Buckinghamshire, UK).

Equal loading of proteins was assessed by stripping blots of bound antibody using ReBlot Plus (Chemicon Internationals, Millipore, Livingstone, UK) for 15 min. (TWIK-2) or 20 min. (TREK-1) at room temperature with gentle agitation. After incubation the stripping solution was removed, blots washed with TBST followed by blocking with 5% Marvel in TBS for 1 hr 30 min. and membranes reprobed with anti-β-actin primary antibody. Protein expression for each sample was normalized relative to the corresponding β-actin signal. For pre-adsorption experiments, anti-TWIK-2 antibody was pre-incubated with the antigenic peptide provided with the antibody at fivefold excess before addition to the membrane blots. The effects of progesterone on TREK-1 protein were studied by incubating Ishikawa cells overnight in the presence of a range of progesterone concentrations (10^−11^–10^−5^ M) and harvesting cells as described above for Western blotting.

### Cell proliferation assay

The effects of K2P channel blockers on cell proliferation were determined using 2500 Ishikawa cells per well of a 96-well microtitre plate, following a 24 hrs serum starvation period. Cells were cultured in DMEM containing 10% stripped serum. Channel blockers, tested in triplicate, were added to the media on day 2 and growth assessed thereafter over a 4-day period. Media and drugs were replaced with fresh substitutes midway through the experimental period. Cell proliferation was monitored daily using the MTS [(3-(4,5-dimethylthiazol-2-yl)-5-(3-carboxymethoxyphenyl)-2-(4-sulfophenyl)-2H-tetrazoilum, inner salt)] assay (Promega, Southampton, UK) at 490 nm. Results from treated cells were compared with control wells to which no test drug had been added. Stock solutions of methanandamide and curcumin were made in 100% ethanol, whereas lidocaine, iberiotoxin, zinc, L-methionine and L-methioninol were dissolved in culture medium. Effects of vehicle alone were tested at concentrations commensurate with those used in the experiment. All experiments were carried out at least twice and samples run in triplicate for each assay.

### Electrophysiology

Freshly dispersed endometrial epithelial cells or cells of the Ishikawa cell line were prepared as described above and allowed to settle for 30 min. on a glass coverslip. Whole-cell voltage-clamp recordings were initiated following formation of a gigaohm (>2 GΩ) seal and capacitance cancellation using filled electrodes with a resistance 4–6 MΩ. Individual cell resting membrane potential was determined under current clamp. Ion currents were elicited thereafter under voltage clamp in response to 10 mV step depolarizations from −60 to +50 mV and current–voltage plots produced. Protocols were delivered through the Axopatch Multiclamp B amplifier with pClamp 9 for acquisition and analysis. The external bath solution consisted of (in mM) 135 NaCl, 5 KCl, 1 CaCl_2_, 1 MgCl_2_, 10 HEPES (pH 7.4), whereas the electrode intracellular solution comprised of (in mM): 140 KCl, 1 KEGTA, 10 HEPES (pH 7.2). Voltage-gated potassium currents were minimized by the addition of 10 mM tetraethylammonium (TEA) and 5 mM 4-aminopyridine (4-AP) to the bath solution. The effects of pH on K^+^ currents were evaluated following acidification of the external bathing solution to the required pH by addition of 0.5 M hydrochloric acid and perfusion of the recording chamber with this modified solution. TEA and 4-AP were purchased from Sigma (Dorset, UK).

### Statistical analysis

Statistical analysis was carried out with GraphPad Prism (V5) software. Data are expressed as mean ± SEM. Results were analysed using non-parametric tests (for qPCR, Western blotting) and Student's unpaired two-tailed *t*-test for electrophysiology. Multiple comparisons of drug treatments for proliferation assays were analysed by anova with post hoc analysis using the Dunnet's test and assigning untreated cells as the control group.

## Results

**Fig. 1:**
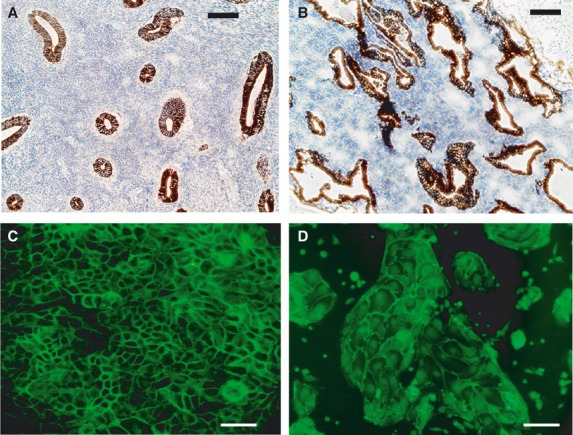
Expression of epithelial cell markers in human endometrium. Freshly collected endometrial samples were formalin fixed for immunohistochemistry or incubated for 1 hr in collagenase to obtain a single cell suspension. For cell culture, epithelial cells were separated from stromal cells by double filtration, plated onto glass coverslips, fixed with paraformaldehyde and then incubated with anti-E-cadherin antibody (AbCam, Cambridge, UK) (**A**) Representative immunohistochemical staining of an early proliferative phase endometrial biopsy. Epithelial endometrial cells express E-cadherin in endometrial glands. E-cadherin immunoreactivity is absent from the stroma and lumen. Scale bar: 100 μm. (**B**) Late secretory phase endometrium. Glands are tortuous in outline and still exhibit E-cadherin staining which is absent from stroma. Scale bar: 100 μm. (**C**) Ishikawa cells at day 8 *in vitro* express E-cadherin at cell–cell boundaries. Scale bar: 10 μm. (**D**) Primary culture of human epithelial cells prepared from a proliferative phase biopsy. E-cadherin immunopositivity is observed at cell–cell boundaries. Scale bar: 5 μm.

Immunohistochemistry of endometrial tissue sections obtained at various stages of the menstrual cycle expressed the epithelial cell marker E-cadherin in glands of both proliferative (*n* = 8) and secretory phase (*n* = 6) endometrium with respective representative images presented in Figure 1A and 1B. Immunostaining was absent from the stroma. Anti-E-cadherin immunofluorescence was also present in primary epithelial endometrial cells cultured for up to 2 weeks and was localized at cell–cell boundaries (Fig. 1C; *n* = 8) as was E-cadherin immunostaining in cells of the Ishikawa cell line (Fig. 1D; *n* = 3).

**Fig. 2:**
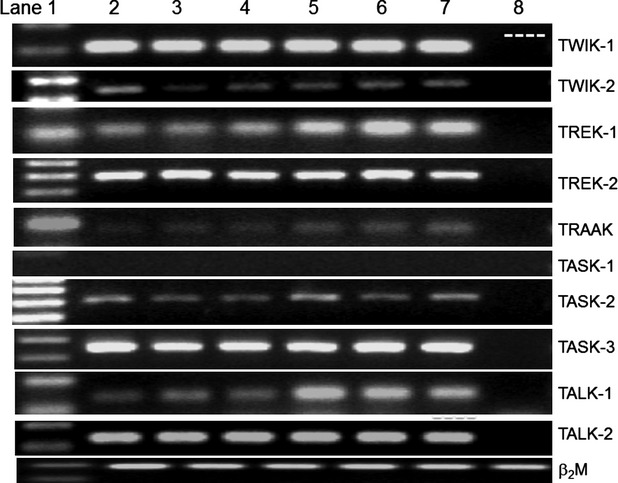
Two-pore Potassium channel expression in the Ishikawa cell line. Conventional end-point RT-PCR was carried out using total RNA extracted from Ishikawa cells (*n* = 3–5) using TriZol. One μg of total RNA was reverse transcribed to produce cDNA. For first strand negative controls, RT enzyme was omitted to check for non-specific amplification. RT-PCR for each individual K2P channel was then performed with relevant primer pairs. The bottom row of the figure shows amplification of the reference gene β_2_M. Ishikawa cells expressed TWIK-1, TWIK-2, TREK-1, TREK-2, TASK-2, TASK-3, TALK-1 and TALK-2. mRNA for TRAAK and TASK-1 channels was low or absent, respectively, whereas the transcripts for TWIK-2 and TASK-2 qualitatively were of low abundance. Lane 1 (L to R) 100 bp ladder. Lanes 2–7 show PCR products from individual preparations of cultured Ishikawa cells. Lane 8 is the no RT control showing an absence of amplification.

**Fig. 3:**
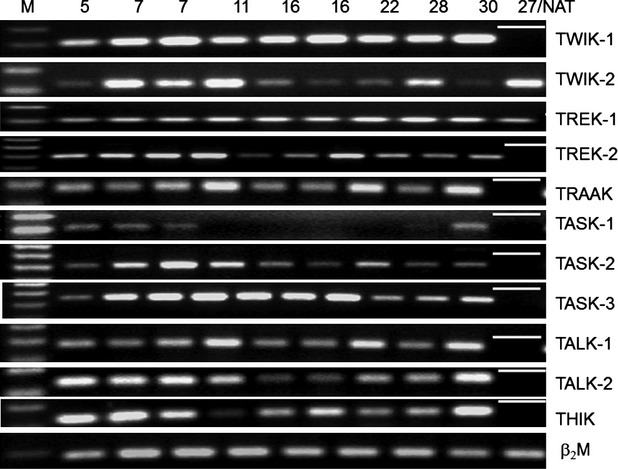
Two-pore Potassium channel expression in human endometrial samples throughout the menstrual cycle. Total RNA was TriZol extracted from 200 mg of snap-frozen endometrium obtained by pipelle biopsy and reverse transcribed into cDNA. Day of cycle of when biopsy was taken is shown above the corresponding lane with days 5–11 considered to be proliferative phase and 16–30 secretory phase endometrium. Expression of TWIK-1, TWIK-2, TREK-1, TREK-2, TRAAK, TASK-2, TASK-3, TALK-1, TALK-2 and THIK was seen in all samples across the menstrual cycle except for TASK-1. Lane 1: 100 bp ladder. The last lane on the RHS depicts either a signal from cDNA for day 27 endometrium (where a band is present) or where no band is present, served as a no added template (NAT) control.

A variety of K2P channel subtypes was expressed in both Ishikawa cells (Fig. 2) and tissue biopsies of human endometrium obtained throughout the proliferative (*n* = 9) and secretory phases (*n* = 7) of the menstrual cycle (Fig. 3). Data from conventional end-point RT-PCR demonstrated mRNA expression of the K2P channel subtypes TWIK-1, TWIK-2, TREK-1, TREK-2, TASK-1, TASK-2, TASK-3, TRAAK, TALK-1, TALK-2 in both Ishikawa cells (Fig. 2) and human endometrium (Fig. 3) producing amplicons of the expected size. Omitting RT enzyme during reverse transcription in the synthesis of cDNA confirmed a lack of non-specific amplification. Comparing K2P expression between cultured Ishikawa cells with endometrial biopsies identified an absence of TASK-1 channel mRNA in the former (Fig. 2) with only low expression apparent in tissue biopsies procured from across the menstrual cycle (Fig. 3). mRNA for TRAAK channels was present in endometrial biopsies, but barely visible in cultured Ishikawa cells (Fig. 3). The reference gene β_2_microglobulin (β_2_M) was expressed in all samples tested (Figs 2 and 3).

**Fig. 4:**
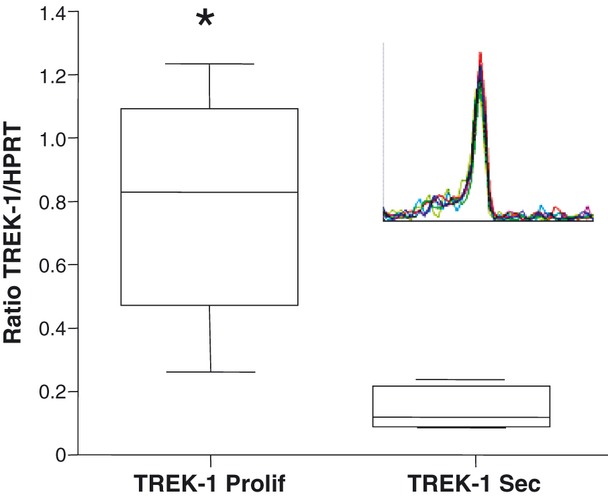
qRT-PCR of TREK-1 mRNA expression across the menstrual cycle in human endometrial biopsies from normal, cycling women (*n* = 14). TREK-1 expression is significantly greater in proliferative (Prolif) *versus* secretory (Sec) phase endometrium (*P* < 0.001). Expression was quantified relative to the reference gene HPRT. Inset shows melt-curve analysis and production of a single, specific transcript from multiple endometrial samples.

**Fig. 5:**
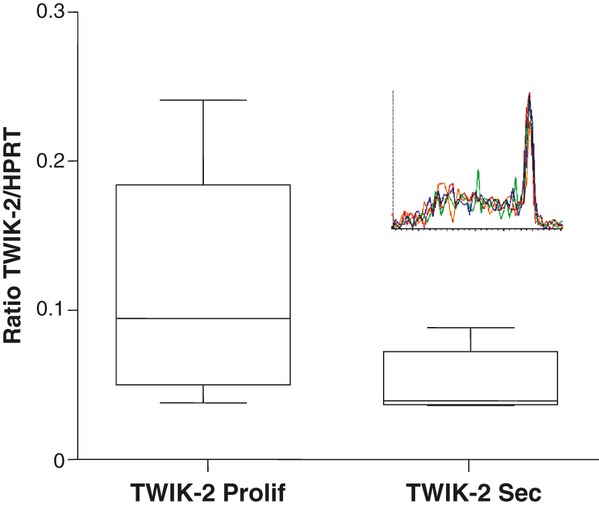
qRT-PCR of TWIK-2 mRNA expression across the menstrual cycle in human endometrial biopsies from normal women (*n* = 14). Expression was quantified relative to the reference gene HPRT. No significant difference (*P* > 0.05) was detected between proliferative (Prolif) and secretory phase (Sec) samples. Inset shows melt-curve analysis of a single, specific transcript from multiple endometrial samples.

Based on the above observations, we elected to quantitate TWIK-2 mRNA expression given the qualitatively distinct sample-dependent changes observed throughout the menstrual cycle (Fig. 3). TREK-1 was also chosen for further investigation on the basis of its apparent constitutive expression (Fig. 3) and multimodal regulation by stretch, lipid, oxygen and pH [10]; factors known to regulate endometrial function. For qRT-PCR experiments, mRNA extracted from whole endometrial biopsies served as template to determine any differential expression of channels with either proliferative or secretory phase endometrium. Expression of four reference genes [β-actin, glyceraldehyde 3-phosphate dehydrogenase (GAPDH), hypoxanthine-guanine phosphoribosyltransferase (HPRT), β_2_M] was examined with no significant difference (*P* > 0.05) amongst them detected across the menstrual cycle. Therefore, HPRT was selected as the reference gene of choice based on its C_t_ values that were closest to those of the target genes being studied. Reactions where reverse transcriptase or cDNA (no added template) was omitted showed no signal amplification. qRT-PCR experiments identified significantly greater (*P* < 0.01) expression of TREK-1 mRNA in endometrial samples harvested from the proliferative (*n* = 8) compared with the secretory phase (*n* = 6) of the menstrual cycle (Fig. 4). The C_t_ values for TREK-1 lay between 28 and 34 with a median of 32 and a median of 26 for TWIK-2 (Fig. 5). TWIK-2 standard curves gave good representative amplification and melt curves (Fig. 5), although levels between proliferative and secretory phase endometrium were not significantly different (*P* > 0.05; Fig. 5).

**Fig. 6:**
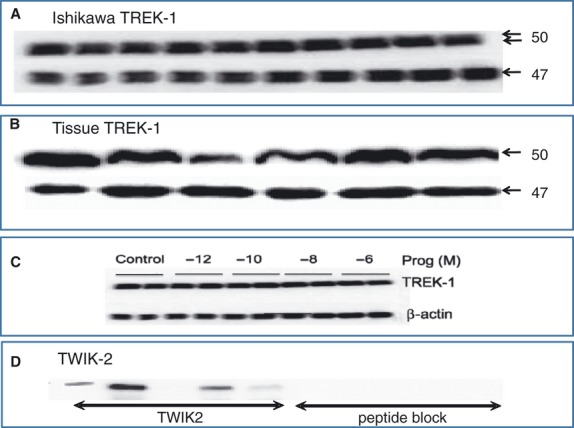
Protein expression of TREK-1 and TWIK-2 in human endometrium. Snap-frozen endometrium and freshly harvested Ishikawa cells were prepared for electrophoresis by homogenization or cell lysis respectively. Proteins were separated by SDS-PAGE using 10% gels followed by electroblotting and incubation overnight with the relevant TWIK-2 or TREK-1 primary antibody prior to chemiluminescent detection. (**A**) Ishikawa cells express TREK-1 seen as a 47 kD band. The lower trace is also a 47 kD band for β-actin that was visualized following stripping and reblotting of the membrane. (**B**) TREK-1 expression in snap-frozen human endometrial biopsies. (**C**) Ishikawa cells incubated at 10^−12^–10^−6^ M progesterone overnight produced no change in signal intensity for TREK-1 compared with control samples. β-actin in the lower trace was used as a positive control. (**D**) TWIK-2 protein was present in human endometrial biopsies. In peptide block experiments where the TWIK-2 antibody was blocked with a fivefold excess of peptide antigen, TWIK-2 signal was abolished (*n* = 3).

Translation of mRNA to protein for TREK-1 was verified by Western blotting in both Ishikawa cells (Fig. 6A) and endometrial biopsies (Fig. 6B; *n* = 9) where a 47–50 kD band corresponding to the monomer of TREK-1 was observed. Owing to the similarities in size of TREK-1 and β-actin, gels were also run separately for each protein without blots being stripped and reprobed. A higher molecular weight ∼100 kD band, likely to be the dimeric form of the channel, was also frequently present (data not shown). The low levels of TREK-1 in proliferative phase endometrium, led us to examine whether progesterone might be driving TREK-1 down-regulation given its critical role in secretory endometrium. Confirmation of oestrogen (ERa and ERb) and progesterone receptor expression in Ishikawa cells was shown by Western blotting (data not shown). Overnight incubation of Ishikawa cells in 10^−12^–10^−6^ M progesterone, corresponding to high circulating levels of the steroid during the late phase of the menstrual cycle, was not associated with a significant down-regulation of TREK-1 protein (Fig. 6C). Expression of TWIK-2 protein was observed as a 37 kD band (*n* = 8), likely to be the TWIK-2 monomer (Fig. 6D). This protein band was absent when the immunoblot was incubated with pre-adsorbed anti-TWIK-2 antibody (*n* = 3; Fig. 6D).

**Fig. 7:**
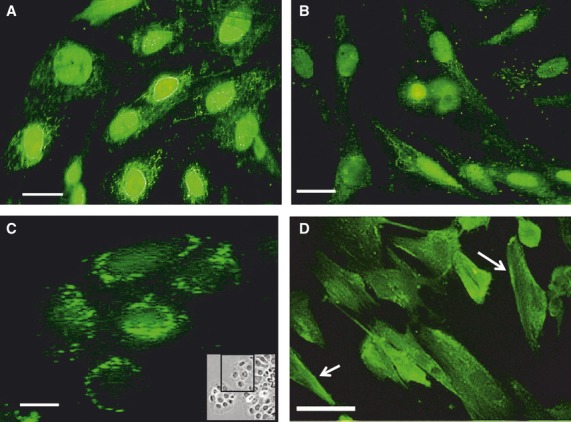
Confocal immunofluorescence of TWIK-2 and TREK-1 in cultured human endometrial cells and Ishikawa cells. Fixation was performed with paraformaldehyde followed by permeabilization then incubation in the relevant primary antibody. Immunofluorescence was localized using FITC-conjugated secondary antibody. Scale bar: 10 μM in (**A**), (**B**) and (**C**), but 20 μM for (**D**). (A) Localization of TREK-1 to intracellular filaments, nuclei and cytosol is clearly evident in cultured endometrium. (B) Ishikawa cells express TREK-1 within nuclei and cytosol. (C) TWIK-2 immunostaining was punctate and membrane bound using Ishikawa cells. Inset shows a bright-filed image of cells sampled. (D) Primary cultures prepared from human endometrium express TWIK-2 on membranes but not nuclei. In some cases, TWIK-2 immunofluorescence was localized to membrane areas not in contact with other cells (white arrows).

Confocal immunofluorescence demonstrated TREK-1 localization to the cell membrane, cytosol and nuclei of both Ishikawa cells (Fig. 7A) and primary cultured epithelial cells (Fig. 7B). Figure 7A also shows the fine, filamentous, intracellular staining present in endometrial cells. Punctate TWIK-2 expression in Ishikawa epithelial cells was membrane-associated in the cell line (Fig. 7C) and primary cultures (Fig. 7D). Interestingly, TWIK-2 immunofluorescence localized to discrete areas located away from cell–cell contacts implying a possible apical location *in vivo*.

**Fig. 8:**
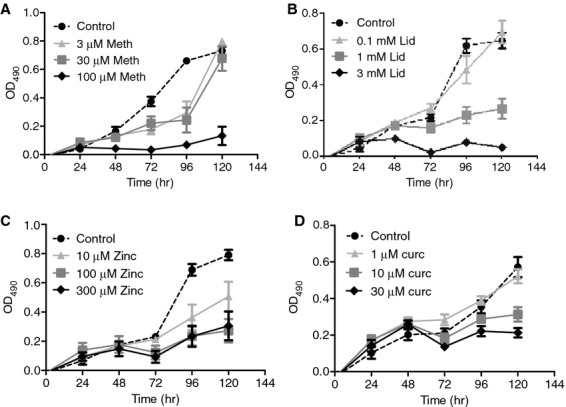
Two-pore Potassium channel blockers inhibited cell proliferation of the Ishikawa cell line. For cell proliferation assays, 2500 cells were incubated in a 96-well plate. Following a serum-starvation period of 24 hrs, drugs were added on the following day and cell proliferation measured as cell viability using the MTS assay, assessed daily at the same time over 5 days. Background absorbance was deducted from all wells and a change in proliferation at various concentrations of drug plotted against absorbance. (**A**) Methanandamide significantly inhibited cell proliferation at 120 hrs by 82%. (**B**) Lidocaine had antiproliferative effects that were observed as early as 72 hrs in 1 and 3 mM. (**C**) Zinc caused dose-dependent inhibition of proliferation by a maximum of 60% at 120 hrs compared with control. (**D**) Curcumin had concentration-dependent inhibitory effects on endometrial proliferation, achieving 44% at 120 hrs.

**Fig. 9:**
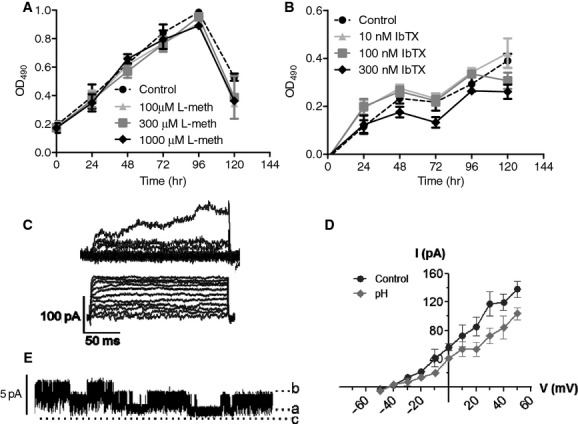
Proliferation and electrophysiology of K2P currents in endometrial epithelia. (**A**) The stretch-dependent channel inhibitor L-methionine had no effect on Ishikawa cell proliferation. (**B**) The maxiK blocker IbTX had only a minimal effect on endometrial cell proliferation. Patch-clamp recordings were made from freshly dispersed and cultured endometrial cells. (**C**) Whole-cell recordings were made in PSS containing 10 mM TEA and 5 mM 4-AP added to the bath to block voltage-gated K^+^ channels to record from K2P channels. Outward currents were apparent for all voltages from −60 to +50 mV. Currents had a rapid onset and were sustained for the duration of the test pulse. Two representative current families are shown from the Ishikawa cells (top) and freshly dispersed endometrial epithelia. (**D**) Reducing pH of the extracellular bathing solution (PSS) from 7.4 to 6.6 produced a reduction at all voltages of outward current in the presence of TEA and 4-AP (*n* = 4). (**E**) Outside-out patches formed after WCR recording typically expressed two types of single channels; a small conductance channel that was constitutively active and characterized by a conductance of 30 pS (*n* = 5) along with a larger channel with a unitary conductance of ∼80 pS (*n* = 4). Both types are present in this representative patch held at a voltage of +50 mV in a quasi-physiological gradient.

Higher potassium channel activation has been implicated in the control of cell proliferation, a key function underlying the endometrial regeneration during the proliferative phase of the cycle when epithelial and stromal cell division is maximal. A panel of drugs shown to inhibit various K2P channels was employed to ascertain effects on cell proliferation. Compared with control, untreated cells, methanandamide (Fig. 8A) and lidoocaine (Fig. 8B) inhibited proliferation, respectively, by 82% and 91% inhibition at 120 hrs *in vitro* in a concentration-dependent fashion. Antiproliferative effects of zinc were apparent at 96 hrs with maximal inhibition of 60% seen at 120 hrs (Fig. 8C). The TREK-1 blocker curcumin reduced proliferation by 44% at 120 hrs at the highest concentration tested (Fig. 8D), whereas L-methionine (Fig. 9A) and L-methioninol, blockers of stretch-dependent channels thought to be TREK-1, showed no effect (*P* > 0.05) on proliferation. As a role for BK_Ca_ channels in proliferation has been reported and given significant single BK_Ca_ channel activity in both Ishikawa and endometrial cells (data not shown), the effects of the BK_Ca_ channel antagonist iberiotoxin (IbTX) were tested demonstrating a small yet significant (<20%; *P* < 0.05) antiproliferative effect observable at 72 and 120 hrs (Fig. 9B).

Whole-cell current-clamp electrophysiological investigations demonstrated that freshly dispersed endometrial epithelial cells were characterized by a mean resting membrane potential of −42.5 ± 5.7 mV (*n* = 9), a value not significantly different (*P* > 0.05) from the −39.3 ± 4.2 mV (*n* = 7) recorded from Ishikawa cells.

Because of the lack of potent and specific blockers of K2P channels, and given the reported insensitivity of several K2P channels to TEA and 4-AP, K2P current was isolated by carrying out whole-cell voltage-clamp experiments in the presence of TEA and 4-AP. The predominant current profiles observed on changing the holding voltage from −60 to +50 mV, consisted of a rapidly activating sustained outward current (Fig. 9C) similar to Ishikawa cells (Fig. 9D) producing a current–voltage relationship exhibiting little rectification. For freshly isolated endometrial cells, when the pH of the extracellular bathing solution was reduced from a pH of 7.4 (control) to 6.6, a significant decrease in whole-cell current amplitude was evident at steady-state at voltages above −10 mV. However, the small sample size meant it was not possible to correlate whole-cell current profiles with cycle-dependent characteristics. Stable outside-out patches were obtained on cessation of whole-cell recording with the majority of patches predominantly silent owing to the presence of TEA and 4-AP in the bathing solution. However, intermittent channel activity characterized by two distinct channel types of 15–25 pS and ∼80 pS was apparent but not characterized further although the smaller conductance channel appeared to be constitutively active.

## Discussion

We report the presence of a variety of K2P channels in human endometrial tissue samples obtained from women of reproductive age and in the Ishikawa endometrial cancer cell line. Our findings demonstrate mRNA expression for the main K2P channel subtypes TWIK, TREK, TASK, THIK and TALK in normal, cycling human endometrium. This finding is consistent with molecular expression of K2P channels in epithelia of taste buds [17], airways [18–20], gastrointestinal mucosa [21] and human nephron [22]. In relation to reproductive physiology, various K2P channels have been reported in sperm [23] and blastocysts [24] where culturing mouse zygotes in media supplemented with K2P blockers or using siRNA to knockdown KCNK expression significantly inhibited mouse blastocyst formation [24]. Virtually undetectable levels of TRAAK expression in the cell line mirror low expression in peripheral organs compared with the brain where TRAAK is highly expressed [14, 25]. In contrast, the presence of TRAAK mRNA in endometrial biopsies was higher than in Ishikawa cells possibly related to TRAAK channels as targets of arachidonic acid in the endometrium where it is a substrate for prostaglandin biosynthesis in bringing about menstruation. Evidence for THIK (halothane-sensitive) channel mRNA is consistent with high copy number of the gene encoding THIK, *KCNK13*, in proliferative phase endometrium [8], but further studies are needed to ascertain its function. The presence of TALK mRNA hints at a potential mechanism whereby the endometrium is able to detect and regulate alkalinity of the reproductive tract. Given that bicarbonate levels are high in uterine fluid and essential for sperm capacitation [26], a combination of TALK and TASK channels will enable the endometrium to respond appropriately to fluctuating pH.

The endometrium is a type of secretory epithelium that undergoes significant changes to both form and function as it progresses through the menstrual cycle suggesting temporal expression of K2P channels. We therefore sought to quantitate TWIK-2 and TREK-1 mRNA levels as the former is expressed in the periphery whereas TREK-1 appears to be modulated by many factors that change throughout the menstrual cycle. The low transcript levels observed for TREK-1 in human endometrial biopsies were not associated with a decrease in TREK-1 protein implying that TREK-1 protein turnover is low as has been noted previously for this channel in odontoblasts [27]. Moreover, TREK-1 expression, evidently abundant in the CNS, is relatively low in peripheral tissues [25]. As endometrial biopsies are heterogenous in composition, consisting of a highly vascularized stroma as well as leucocytes, it is difficult to identify with certainty the nature of the cells expressing TREK-1. It should be noted that progesterone regulation of TREK-1 channels was assessed in the Ishikawa cell line and although it may be a suitable experimental model in which to study epithelial function, this cell line does not replicate characteristics of normal, cycling endometrium and is therefore of limited value.

Endometrial TREK-1 protein was identified as a 47 kD monomer consistent with its predicted band size from earlier observations [28–30], but is at variance with reports of a 90 and 60 kD TREK-1 protein in reproductive tissues [31, 32]. In common with its localization in myometrium [31], placental cytotrophoblasts [32], endothelium [33] keratinocytes [34] and prostate cancer cells [35], immunofluorescence for endometrial TREK-1 was observed in the cell membrane, cytoplasm and nuclei. This could be because of the lack of commercial antibodies with high specificity for the TREK-1 epitope although TREK-1 staining has been linked with axonal transport and protein trafficking [28]. Voloshyna *et al.,* propose that the nuclear localization observed when TREK-1 is overexpressed in prostate cancer influences cell proliferation [35]. Our finding that progesterone did not lead to reduced TREK-1 expression implies additional regulatory mechanisms governing molecular expression of this channel. Recently, a truncated TREK1 splice variant, TREK1ΔEx4, has been shown to interfere with the trafficking of wild-type TREK-1 to the membrane, thus causing a functional down-regulation manifest as a decrease in TREK-1 current [36]. Although determined in the brain, this mechanism of alternative translation initiation adds to other postulated mechanisms that use K^+^ channel binding partners to regulate trafficking and surface expression of K2P channel subunits and requires investigation in non-excitable cells.

No evidence from our study was found to support differential TWIK-2 mRNA expression between the proliferative and secretory phases. Davis and Cowley [18] have shown apical TWIK-2 channel expression in human airway Calu-3 cells where the channel facilitates anion secretion. The cellular localization of TWIK-2 to the apical surface has also recently been confirmed in normal human bronchial epithelium where blockade of TWIK-2 channels with either bupivacaine or quinidine inhibited sodium absorption through ENaC and forskolin-induced anion secretion [20]. Although the culture model used in our study did not maintain epithelial cell polarity, culturing cells directly on a substrate that faithfully replicates 3D structure, in parallel with studies of short-circuit current flow, would aid in clarifying the role of TWIK-2 in relation to electrolyte transport across apical and basolateral aspects of endometrial epithelia.

Opening of K^+^ channels underlies cell volume changes accompanying proliferation by mediating the progression of various cell types from the G1 to the S phase of the cell cycle enabling cellular hyperpolarization through K^+^ efflux [37, 38]. Despite the presence of a multitude of mammalian voltage- and ligand-gated K^+^ channels, only TASK-3 [39] and TREK-1 [35, 40] of the K2P channels have definitively been linked to cell proliferation. The inhibition of endometrial epithelial cell proliferation by the K2P channel blockers lidocaine [41], methanadamide [42] and zinc [43] point to the involvement of TASK channels in the proliferative process given their oncogenic potential [39, 44]. The inhibitory effects of methanandamide through TASK blockade do not exclude antiproliferative effects *via* endometrial cannabinoid receptor blockade [45], although no change in CB1 or CB2 receptor density with the menstrual cycle has been noted [45]. Nor is there any evidence to date to support cannabinoid receptor involvement in endometrial proliferation. Zinc has dual effects, blocking TASK-2, TASK-3 and TWIK-2 channels yet also activates TREK-1 and TREK-2 [43]; with these opposing effects, it is difficult to discern the precise target of action of this divalent cation. The action of lidocaine would likely be through TASK-2 channels that are categorized as alkaline sensitive and exhibit properties similar to TALK channels.

Endometrial cell proliferation was also reduced by curcumin which inhibits bovine TREK-1 channels resulting in cortisol secretion from the adrenal gland [46]. Curcumin has an antiproliferative effect in prostate cell lines [47] with possible links to prostate cancer [35]. Thus, we propose that it is likely that TASK channels, but possibly also TREK-1, have a role to play in endometrial epithelial cell proliferation. An anti-apoptotic role for TREK-1 [48] which would favour a proliferative phenotype, consistent with the process of endometrial regeneration following menstruation, has been postulated. Indeed, apoptosis increases in the endometrium during the late secretory phase [49] where we also determined mRNA levels for TREK-1 to be at their lowest. Hyperpolarization in several cell types is associated with mitogenic effects mediated *via* G1/S cell-cycle progression [37] and is a potential function of K2P channels, recognized for their role in the generation of a background, leak current. Linking membrane potential through K2P channels with proliferation and/or apoptosis is complex and influenced by many factors that likely include intracellular calcium and chloride concentrations, cell-cycle stage and checkpoint proteins (*e.g*. cyclins). The nuclear localization of TREK-1 that we and others [35] have reported is intriguing and does not exclude indirect TREK-1 modulation of molecular mechanisms, notably gene transcription with subsequent downstream effects on cell proliferation.

Two-pore potassium channels are notoriously difficult to study in native cells given the lack of pharmacological agents that lack the specificity to dissect channel function but also the possibility of redundancy whereby leak channels may be able to compensate for others owing to their shared functions.

A recent report has implicated BK_Ca_ channels in endometrial receptivity and embryo implantation based on reduced rates of implantation, and down-regulation of factors such as nuclear factor-κB, induced by knockdown of the BK_Ca_ channel [50]. We observed only a small but significant role for BK_Ca_ in cell proliferation, although our single-channel recordings have shown the BK_Ca_ to be one of the most active channels in endometrial epithelium (RN Khan, unpublished observations).

The regulation of endometrial whole-cell currents by extracellular pH is an interesting phenomenon. In the female reproductive tract, pH of human uterine fluid, produced by endometrial epithelia fluctuates between 6.6 and 7.6 and is stage-dependent, tending towards acidic around the time of ovulation [51]. Following coitus, uterine fluid is rendered alkaline which is conducive to maintaining sperm motility while a low pH is hostile to sperm activation and therefore conception. In our experiments, acidic pH reduced the whole-cell outward K^+^ conductance at all voltages which would tend to depolarize the cell. As our protocol blocked voltage-gated channels and only TASK channels of the K2P superfamily are responsive to extracellular acidic pH, we postulate that TASK channels are putative sensors linking pH with membrane potential in the endometrium. Gardener *et al.,* [33] working with rat mesenteric and pulmonary arteries argue in favour of TASK-1 channels mediating the membrane potential response to pH. The conductance of the channel we observed is within the range of values reported for K2P channels [10] although further electrophysiological characterization of these channels is necessary before they can be identified definitively as belonging to the K2P family. Although these data are preliminary, the fact that endometrial epithelial cells generate outward currents that have a sustained activation, display sensitivity to pH and are not blocked by TEA or 4-AP suggests that these currents are carried by K2P channels and may have an important physiological function in the setting of endometrial cell membrane potential.

The endometrium is unique in terms of the sheer magnitude and frequency of proliferation it undergoes through a woman's reproductive life solely in anticipation of supporting pregnancy. Although we exercised great care in only including patients with a clear menstrual history that met our criteria, future, detailed investigations of ion channel expression and regulation will require sampling of endometrial biopsies with greater rigour. For example, we assigned samples to either proliferative or secretory phase. In reality, these stages are further divisible into early, mid and late stages based on histological dating of endometrial biopsies. Analysis in this sequential fashion will allow us to determine more robustly the role of K2P channels in cellular and molecular mechanisms linked to reproductive events and their potential utility in the management of fertility.

In terms of the clinical relevance of our findings, endometrial thickness, measured by ultrasound, is arguably, one of the most reliable indicators, to date, of successful pregnancy outcome following IVF [52]. Our data indicating that blockade of TASK and TREK channels inhibits proliferation may underlie some cases of female infertility where an inadequate (thin) endometrial lining may arise *via* dysfunction or aberrant expression of K^+^ channels such that cell division and endometrial remodelling is suboptimal consequently proving detrimental to implantation. Moreover, altered membrane potential responses induced by acidification in normal endometrium reported here and, considering the impact of pH and ionic gradients in the female reproductive tract on fertilization rate [4], we suggest that the acid-base balance of the female reproductive tract is an important determinant of pregnancy rates. To our knowledge, with the exception of fertility treatments where embryo quality is optimized through tight control of pH and culture conditions, investigations of pH shift with the menstrual cycle, and correlation with reproductive outcome have not been performed yet constitute relatively simple measures to explore unexplained infertility. As a longer term clinical goal, our findings highlight the prospect of endometrial ion channels as potential non-steroidal contraceptive drug targets. In summary, our data provide new evidence for TREK-1 and TASK channel expression and their roles in endometrial epithelial cell proliferation. Evidence in favour of a contribution of TREK-1 in proliferative phase endometrium is corroborated by its higher expression during this period and our finding that the TREK-1 modulators curcumin and zinc modify cell proliferation. Lidocaine, zinc and methanandamide as inhibitors of TASK channels is further proof of K2P channel involvement in proliferation. Direct pH regulation of K^+^ currents in endometrial epithelia implies that either TASK-1 and/or TASK-3 channels mediate this response. The control of fertility remains an important goal in reproductive medicine. Despite celebrated advances in reproductive technologies, our meagre understanding of the human endometrium and the fact that idiopathic infertility accounts for over one third of infertility will benefit from detailed analysis of endometrial K2P channels and their clinical application as potential targets through which to modify fertility and reproductive outcome.
